# Titer improvement of mycophenolic acid in the novel producer strain *Penicillium arizonense* and expression analysis of its biosynthetic genes

**DOI:** 10.1186/s12866-023-02884-z

**Published:** 2023-05-17

**Authors:** Hala A. Ammar, Saeid M. Ezzat, Ebrahim Elshourbagi, Hind Elshahat

**Affiliations:** 1 Botany and Microbiology Department, Faculty of Science, Zagazig University, Zagazig, Egypt; 2 Animal Health Research Institute, Cairo, Egypt

## Abstract

Mycophenolic acid (MPA) is the active ingredient in the most important immunosuppressive pharmaceuticals. It has antifungal, antibacterial, antiviral, anti-psoriasis, and antitumor activities. Therefore, its overproduction in addition to gene expression analysis was our main target. Through this study, we isolated a novel potent mycophenolic acid (MPA) producer strain of the genus *Penicillium* from the refrigerated Mozzarella cheese and it was identified with the molecular marker ITS and *benA* genes as *P. arizonense*HEWt1. Three MPA overproducer mutants were isolated by exposing the wild type to different doses of gamma-rays, and the fermentation conditions for the highest production of MPA were optimized. The results indicated that MPA amounts produced by the mutants MT1, MT2, and MT3 were increased by 2.1, 1.7, and 1.6-fold, respectively, compared with the wild-type. The growth of both mutant and wild-type strains on PD broth, adjusted to pH 6 and incubated at 25 °C for 15 d, were the best conditions for maximum production of MPA. In a silico study, five orthologs genes of MPA biosynthesizing gene clusters in *P. brevicompactum* were predicted from the genome of *P. arizonense*. Sequencing and bioinformatic analyses proved the presence of five putative genes namely *mpaA, mpaC, mpaF, mpaG*, and *mpaH* in the *P. arizonense* HEWt1 genome. Gene expression analysis by qRT-PCR indicated an increase in the transcription value of all annotated genes in the three mutants over the wild type. A highly significant increase in the gene expression of *mpaC, mpaF*, and *mpaH* was observed in *P. arizonense*-MT1 compared with wild-type. These results confirmed the positive correlation of these genes in MPA biosynthesis and are the first report regarding the production of MPA by *P. arizonense.*

Kew word.

Mycophenolic acid, *Penicillium arizonense*, mutagenesis, gene expression.

**Supplementary Information:**

The online version contains supplementary material available at 10.1186/s12866-023-02884-z.

## Introduction

The wide metabolic variety within the fungal kingdom continues to supply an opulent source for novel drugs. The physiological and chemical diversity in the fungal kingdom leads to the identification of novel bioprocesses depending on fungi that can meet the demands of the current society for creative pharmaceuticals and antimicrobials. Huge numbers of secondary metabolites were previously detected in the fungal kingdom. However, the evolution of the discovery of proper bioactive secondary metabolites to their applications remains a challenge. *Penicillium* is a diverse genus distributed worldwide in various habitats. Although DNA sequences are necessary for the strong identification of different species of *Penicillium*, there is currently no universal reference database for verification of its genus. *Penicillium* genera contain more than 354 accepted species [1], which are a vital industrial unit for the biosynthesis of many chemically and structurally diverse bioactive secondary metabolites, including significant pharmaceutical compounds [2, 3]. However, the representation of fungal kingdoms in industrial bioprocesses is still limited, and the improvement of the production of significant compounds is considered an intensive challenge and remains expensive. Mycophenolic acid (6-(4-hydroxy-6-methoxy methyl 3oxophthalanyl)-4-methyl-4-hexenoic acid, C17H20O6 (MPA) is a famous secondary metabolite [4] that was discovered as a product of *Penicillium brevicompactum* before the start of the 20th century. Some *Penicillium* species have also been reported to produce it, including *P. brevicompactum*, *P. viridicatum, P. stoloniferum* and *P. roquefourti* [5–7]. Mycophenolic acid and its precursors, 5-methylorsellinic acid and 5,7-dihydroxy4-methylphthalide, were also identified as secondary metabolites of the yeast *Byssochlamys nivea* [8]. Due to its immunosuppressive and antimicrobial properties, it has been characterized as an important bioactive compound with various biomedical applications [9]. Additionally, MPA has regular medical applications as an immunosuppressive drug in the form of mycophenolate mofetil, which has been used to treat different autoimmune diseases [4, 10, 11]. Much interest has been directed to the industrial production of MPA due to its potent antiviral effects [12, 13]. Recent studies proved that MPA has an effective antiviral activity against MERS-CoV, human coronavirus (HCoV)‐OC43, HCoV‐NL63, and SARS‐CoV‐2 [14–16], and reported that the combination of MPA and interferon can be used for the treatment of COVID-19. For this intensive importance of MPA, it is necessary to search for a new *Penicillium* species to produce MPA and to improve its productivity through the batch fermentation process. Cheese is a favorable substrate for the growth of various species of *Penicillium* because of its favorable preservation conditions for fungal growth, such as low temperature and moisture content. Previous studies reported the distribution of *Penicillium* species in a wide range of refrigerated cheeses [17]. Although it seems to be no record of the appearance of harmful effects in humans following the consumption of fermented blue cheese, scientific research has increased attention on the production of some secondary metabolites by *Penicillium* species isolated from these cheeses. A known metabolite of *Penicillium* connected with blue cheeses is mycophenolic acid (MPA) [18, 19]. Therefore, it was used as a source for the isolation of a new MPA-producing species belonging to the *Penicillium* genus.

The molecular basis of MPA biosynthesis was recently investigated in both *P. brevicompactum* and *P. roqueforti* [6, 20–22]. The MPA gene cluster in *P. brevicompactum* consists of 8 open reading frames (*mpaA, mpaB, mpaC, mpaD, mpaE, mpaF, mpaG, and mpaH)*, which encode a putative prenyltransferase, a protein with unknown function, a polyketide synthase, a natural fusion of a cytochrome P450 domain, a hydrolase domain, an inosine-5´-monophosphate dehydrogenase (IMPDH), an O-methyltransferase, and an oxidative cleavage enzyme, respectively [6, 20, 21]. The *mpaC* is responsible for the first step in MPA biosynthesis through the production of 5-methylorsellinic acid (5-MOA, which is encoded by polyketide synthase) [6]. Moreover, it was recently illustrated that the *mpaF* gene encodes an MPA-insensitive inosine-5´-monophosphate dehydrogenase (IMPDH), which confers self-resistance toward MPA [20, 23]. The investigation of the molecular basis of MPA production in producer fungal species has a significant impact on facilitating the understanding of the mechanism of its production and controlling how it can be improved, especially when the producer species is the first record. The gene cluster of bioactive metabolites typically consists of the genes that confer the needed toleration on the compounds. MPA is an inhibitor of IMPDH, which is responsible for the conversion of IMP to XMP in the novel pathway of GMP biosynthesis [24]. This reaction is important for all living microorganisms. Inosine-5’-monophosphate dehydrogenase (IMPDH) genes are highly conserved among various species of microorganisms.

*P. arizonense* is a strong producer of various secondary metabolites and different proteins involved in carbohydrate metabolism. Large numbers of secondary metabolites, such as tryptoquivalines, pyripyropenes, austalides, fumagillin, pseurotin A, xanthoepocin, and curvulinic acid, were investigated in the cell extract of *P. arizonense* [3]. Genome sequencing analysis of *P. arizonense* was assembled into 33.7 Mb containing 12,502 predicted genes [3]. Between these genes, 62 putative biosynthetic gene clusters are involved in lipid metabolism and secondary metabolite production. Up till now, there have been several incompletely annotated open reading frames (ORFs) in the *P. arizonense* genome. For these reasons, we aimed to overproduce MPA by a novel producer species of *Penicillium*, namely, *P. arizonense*, and to study the expression of the MPA-producing genes through identification and cloning of the synthesizing genes of *P. arizonense* that are orthologous to the MPA biosynthesizing gene cluster in *P. brevicompactum*. We also focused on studying the gene expression of the identified genes in combination with a physiological analysis of MPA production. There is no previous study on the phenotypic and genotypic analysis of MPA production by *P. arizonense*.

## Materials and methods

### Chemicals

All chemicals used in this study were purchased from Sigma Chemical Co. (St. Louis, MO). The stock solution of MPA was prepared by dissolving 1 mg/mL mycophenolic acid in methanol and stored in the dark at 4 °C.

### Culture media

Six types of culture media were used in this study. Czapek-Dox-Yeast extract medium (CDY) was composed of 20 (g/l): sucrose 30, NaNO_3_ 3, KH_2_PO_4_ 0.5, KCl 0.5, MgSO_4_.7H_2_O 0.5, and 0.5 yeast extract FeSO_4_.7H_2_O 0.01 (g/l). Yeast Extract-Sucrose medium (YES) composed of (20 g/l): sucrose 50, yeast extract. Potato-Dextrose medium composed of (g/l): peeled potato slices 200, glucose 20. Yeast Peptone Dextrose (YPDA) was composed of (g/l): 10.0 g yeast extract, 20.0 g peptone, 20.0 g dextrose, and 20.0 g. Malt Extract Sucrose (MES) medium was composed of (g/L): 150.0 sucrose, 20.0 malt extract, 0.5 MgSO_4_.7H_2_O. Glucose Peptone medium (LCG) composed of (g/L): 150.0 D-Glucose, 5.0 peptone, 1.0 K_2_HPO_4_, 0.5 MgSO_4_.7H_2_O, 5.0 NaCl and 1000 mL distilled H_2_O.

### Maintenance of MPA producer strain

The MPA producer fungal strain was isolated from Mozzarella cheese located in Egyptian markets. The wild type, *Penicillium arizonense*, and the mutants used in this work were preserved on potato dextrose agar (PDA) slants at 4 °C. The wild type-strain was deposited in the public culture collection, belonging to the WDCM, of Ain Shams University and registered under the number CCASU-2022-F4.

### Morphological and molecular identification of isolated Fungi

The purified isolates were sub-cultured on Potato Dextrose Agar medium (PDA) and allowed to grow at 25 °C for 7 days. Morphological identification was performed according to **Raper and Fennell** [25] and **Pitt and Hocking** [26]. The MPA producer strain was identified morphologically according to microscopic characterization as well as molecular analysis. After 7–10 days, some colony properties such as color, texture, and diameter of conidia describing the morphology of *Penicillium* species, and the degree of sporulation were considered [1]. The characteristics of colonies were recorded and compared with the identification keys [27]. The arrangement of conidia, the intact fruiting body including hyphae, and the phialides were observed after their staining with lactophenol cotton blue (LCB) [28]. Molecular identification of the experimental MPA producer strain was also analyzed, using the internal transcribed spacer (ITS) and the *benA* genes, to confirm the morphological identification according to **Visagie et al.** [1]. Genomic DNA was extracted from 3-day-old cultures according to the CTAB method [29, 30].

The fungal isolate was identified by ITS rDNA sequencing analysis (18 S, 28 S rRNA, flanking ITS 1, 5.8 S rRNA, and ITS 2) according to **White et al.** [31]. The molecular identification of the wild-type strain was carried out using the molecular marker β-tubulin (*benA*) gene, in addition to the ITS gene, according to **Visagie et al.** [1]. The primers Bt2a and Bt2b were used for the amplification of *benA* [32]. PCR analysis was carried out according to **Sambrook and Russell** [33]. The released sequences were deposited in the GenBank database, and Molecular Evolutionary Genetic Analysis (MEGA version 7) software was used for phylogenetic analyses [34]. The closest homologous sequences were selected, and multiple sequence alignments were carried out using the ClustalW program in MEGA7 software. The initial tree(s) for the heuristic search were obtained automatically by applying the Neighbor-Join and the BioNJ algorithms to a matrix of pairwise distances estimated using the Maximum Composite Likelihood (MCL) approach and then selecting the topology with the superior log likelihood value [35]. Evolutionary analyses were conducted in Mega7 software.

### Estimation of MPA

#### Thin-layer chromatography analysis (TLC)

MPA was determined qualitatively using TLC. The methanol extract samples were loaded with the reference standard of MPA on TLC plates using the solvent system toluene: ethyl acetate: formic acid (6:3:1, v:v:v). MPA, with an Rf value of 0.65, showed blue fluorescence spots under long-wavelength ultraviolet light. The spots were visualized by exposing the plates to ammonia vapor before observation under ultraviolet light. The fluorescent spots that appeared identical to the authentic MPA were scraped off and eluted with chloroform. MPA was then quantified by ultraviolet spectroscopic analyses performed with a T80 + UV Flash spectrophotometer PG Instrument LTD, UK spectrophotometer, UK. MPA absorption was measured at 304 nm, and the concentration was obtained after recording the optical density against a standard curve [36].

#### High-performance liquid chromatography (HPLC) analysis

The purified MPA was further analyzed using high-performance liquid chromatography (HPLC) using HPLC, EZChrom Elite Client/Server, Agilent, USA [37]. The stock solution of MPA (1 mg mL^− 1^) was prepared in methanol and stored at 20 °C. The working solution of MPA in the range of 10–100 µg mL^− 1^ was prepared by serial dilution of the stock solution with methanol.

### Inducing mutagenesis

The irradiation process was carried out by exposing the wild-type *P. arizonense* strain to different gamma rays at the National Center for Radiation Research and Technology (NCRRT), Nasr City, Cairo, Egypt. The fungal spore suspensions were exposed to various doses of ^60^Co γ-rays (150, 200, 250, 500, and 750 Gy) emitted by an Indian gamma-ray device through an Indian gamma cell. The irradiated spores were kept in the dark to prevent any photoreaction. The treated and untreated spores were inoculated onto PDA plates by the serial dilution method and incubated at 25 °C for 3 days. Fifty single colonies were selected according to the variation in the color of the metabolite, and the shape and color of the colonies. The selected mutants were preserved in the dark at 4 °C. The mutants were inoculated into MPA production medium and incubated for 10 days at 25 °C. The MPA productivity of the selected mutants was quantitatively estimated, and the highest producer mutants over the wild type were used for stability examination. The stability of each mutant was tested for four generations according to **Luthra et al.** [38], and the three most stable mutant strains were selected for our study.

### Optimization of fermentation conditions

The fermentation conditions, including culture media, incubation temperature, initial pH values, and fermentation periods, were screened for maximum production of MPA by both the WT strain, *P*. *arizonense* HE-MPw1, and the highest producer MT, *P*. *arizonense* HE-MPM1. Six types of broth cultures (PD, CDY, YES, LCG150, MES, and YPD) were screened for optimal production of MPA. The incubation time was selected between 10 and 21 days, the tested temperatures were adjusted in the ranges of 20 and 40 °C, and the pH values were adjusted in the ranges of pH 2–8 using citrate phosphate buffer [39]. One milliliter of approximately 10^7^ freshly prepared 5-day-old spore suspensions of the WT and the MT strains was inoculated into 50 mL of sterilized MPA producer medium and incubated under selected conditions according to the experimental design. At the end of the incubation time, the mycelial dry weight, and the amount of MPA were evaluated.

### Nucleic acid extraction

Genomic DNA was extracted using the CTAB method as mentioned above. RNA was extracted by the Triazole reagent protocol (Invitrogen, Carlsbad, CA, USA) according to the manufacturer’s instructions. The extraction protocol was modified by some additional steps according to **Sah et al.** [40]. The RNA pellet was dissolved in 60 µL of autoclaved DEPC-treated water and stored at -20 °C until use. The primers of the predicted genes, orthologous to MPA biosynthesizing MPA, were designed from the orthologous coding sequence of the MPA gene cluster (*mpaA, mpaC, mpaF, mpaG* and *mpaH*) of the *P. roqueforti*, and *P. brevicompactum* genomes.

### Bioinformatic analysis of the MPA genes and primers design

The MPA gene cluster was identified in the BGC, antiSMASH, and MIBiG databases through the secondary metabolite bioinformatics portal https://mibig.secondarymetabolites.org/. The high conservation between the MPA biosynthesizing genes *mpaF*, *mpa*C, and *mpa*G of *P. brevicompactum* and their orthologous genes in the *P. arizonense* genome encouraged us to scan the whole similar putative protein that is responsible for MPA production in the genome of *P. arizonense* using BlastX, BlastP and BlastN databases. One contig (GenBank accession numbers LXJU01000010.1) containing Open Reading Frames (ORFs) encoding significant similar proteins to *mpaA, mpaC, mpaF* and *mpaH*, and another contig (GenBank accession numbers LXJU01000005) encoding significant similar protein to *mpaG* were identified and downloaded. Blast searches were carried out using the online web link http://blast.ncbi.nlm.nih.gov. Because these genes play an important role in MPA biosynthesis, we analyzed their expression related to the production of MPA in both wild-type and mutant strains. The primers for the MPA biosynthesizing genes were designed from both *P. brevicompactum* MIBiG accession BGC0000104 and NCBI GenBank: HQ731031.1 and they were aligned with their homologous sequences of *P. arizonense* (strain: CBS 141,311). All primers were designed by the Primer 3 plus program http://primer3plus.com/cgi-bin/dev/ primer3plus.cgi and were listed in Table S. 1. The primers were tested for amplification of their corresponding fragments by amplification of conventional PCR analysis. The amplicons were identified using agarose gel electrophoresis. The designed primers were purchased from Sigma-Aldrich (St. Louis, MO).

### Detection of MPA genes in the *P. arizonense* genome using conventional PCR analysis

The wild type, *P. arizonense-*HEwt, and the three MPA producer mutants were inoculated into PD broth and incubated at 25 °C for 5 days under shaking conditions at 150 rpm. MPA was determined as shown previously, and mycelial wet mats were used for DNA extraction. Conventional PCR amplification of the five MPA genes of wild-type and mutant strains, was carried out using the designed oligonucleotide primers shown in Table S. (1). PCR was performed in a final volume of 20 µL at the following reagent concentrations: 4 µL of 5× Phusion HF Green buffer, 0.4 µL of 10 mM dNTPs, 10 pmol of forward and reverse primers (1 µL each), Phusion 0.2 µL of HF DNA polymerase enzyme at 2 U/µL, 2 µL of template DNA (approximately 10 ng), and the total volume of the PCR mixture was adjusted to 20 µL with nuclease-free water. PCR amplification included an initial denaturation step at 98 °C for 30 s, followed by 35 cycles at 98 °C for 10 s, an annealing step for 15 s at 60 °C, an extension step for 20 s at 72 °C, and a final extension for 5 min at 72 °C. The PCR products were analyzed by 1% agarose gel electrophoresis in 1× TEA buffer at room temperature. For gel analysis, 5 µL of the PCR products were loaded in each gel slot. A 100 bp DNA Ladder (Qiagen) was used to determine DNA fragment size. The gel was photographed by a gel documentation system.

### Gene expression, sequencing, and phylogenetic analysis

Quantitative real-time PCR (qRT-PCR) analysis was used to estimate the gene expression of the predicted MPA genes in *P. arizonense*. The wild-type and producer mutant strains were inoculated in PD broth and incubated for 5 and 10 days under favorable conditions for MPA production. After the incubation time, mRNA was extracted from the fungal mats, and the cDNA of the five genes was transcribed using reverse transcriptase enzyme using the same primers mentioned previously. The primers were designed for gene coding sequences for partial amplification of cDNA. Primers were utilized in a 25-µL reaction containing 0.25 µL Verso Enzyme Mix (including RNase inhibitor), 12.5 µL 2× Quanti Tect SYBR Green PCR Master Mix (Qiagen), 1.25 µL RT enhancer (Thermo Scientific), 0.5 µL of each primer (10 pmol µL^− 1^), 2.5 µL cDNA template and 6.5 µL water. The reaction was performed in a Strata-gene MX3005P real-time PCR machine. PCR conditions were as follows: reverse transcription at 50 °C for 30 min, initial denaturation for 5 min at 94 °C, followed by 40 cycles of secondary denaturation at 94 °C for 15 s, annealing at 54 °C for 1 s, and extension at 72 °C for 45 s, followed by 1 cycle for dissociation curve analysis of denaturation at 94 °C for 1 min, annealing at 54 °C for 1 min, and final denaturation at 94 °C for 1 min. Amplicons size for each primer pair was verified by gel electrophoresis. The *β-actin* gene was used as a housekeeping gene through two primers: ACT 512 and ACT 783 [41]. The qRT-PCR data were analyzed by IQ5 optical system software (BioRad, Hercules, CA). The threshold cycles were calculated using the PCR baseline subtracted mode, and the amplification efficiency for each gene amplified from wild-type and mutant strains of *P. arizonense* was estimated. CT values and amplification curves were calculated using Stratagene MX3005P software (Stratagene, La Jolla, CA). The CT value of the *β-actin* gene, derived from the amplicons, was used as a reference for normalization. To determine the variation in the expression of the MPA genes from the mutant strains, the CT value of each sample was compared with that of the positive control (wild-type strain) according to the BΔΔCt^ method reported by **Yuan et al.** [42]. The purified PCR products of the MPA genes, amplified from the wild-type strain of *P. arizonense*, were sequenced in the forward and reverse directions on an Applied Biosystems 3130 Automated DNA Sequencer (ABI, 3130, Applied Biosystems, Foster City, CA) using a ready reaction Bigdye Terminator V3.1 cycle sequencing kit (Perkinelmer/Applied Biosystems; Cat. No. 4,336,817). A BLAST® analysis, Basic Local Alignment Search Tool [43], was initially performed to establish sequence identity to GenBank accessions. The retrieved sequences of all genes were annotated and aligned through the NCBI database using the online website link http://blast.ncbi.nlm.nih.gov. The completely annotated sequences were registered in the GenBank database under the accession numbers listed in Table S. (2). Multiple sequence alignments of the deduced DNA and proteins were analyzed using the ClustalW program in MEGA7 software, and phylogenetic analysis of both protein and DNA sequences was carried out. The evolutionary history was inferred using the neighbor-joining method [44]. The evolutionary distances were computed using the p-distance method and are in units of the number of amino acid differences per site. Evolutionary analyses were conducted in MEGA7 [35]. The conserved regions of the identified MPA genes were analyzed for their related protein families in the conserved domain database https://www.ncbi.nlm.nih.gov/cdd/.

### Statistical analysis

Statistical analysis was performed using SPSS version 25 (IBM Corp., Armonk., NY). Data were presented as the mean ± standard deviation (SD). For all experiments, a minimum of 3 biological replicates were used. The paired samples t-test was used for the comparison of the mutants (after 5 days and 10 days of incubation), while one-way analysis of variance (ANOVA) with post hoc pairwise comparisons adjusted by Tukey’s post-test was performed for the evaluation of the difference between mutants in each gene. *P* values less than 0.05 were considered statistically significant.

## Results

### Identification of a novel MPA producer *P. arizonense*

**Fig. 1:**
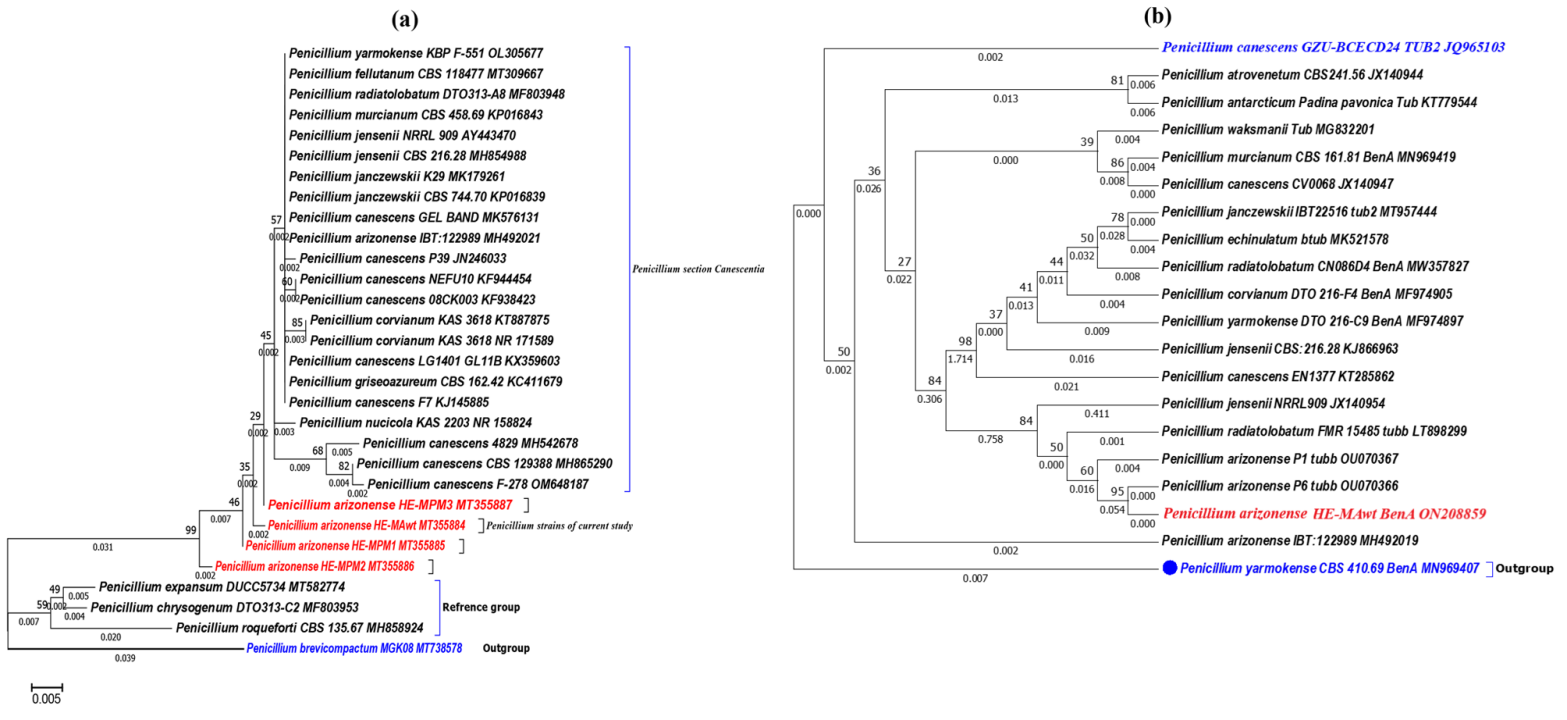
Phylogenetic analysis of *P. arizonense* HE-MAwt (MT355884) using two different genetic markers, (**a**) ITS and (**b**) *ben A* barcode. The phylograms show the relationship of *P. arizonense* HE-MAwt with the closely related strains of the same species, retrieved from NCBI GenBank database, and its relation to the *Penicillium* species of section *Canescentia*. The percentage of replicate trees in which the associated taxa clustered together in the bootstrap test (1000 replicates) is shown next to each branch. The tree is drawn to scale, with a branch length in the same unit as those of the evolutionary distance used to infer the phylogenetic tree. The evolutionary distance was computed using the Maximum Composite Likelihood method and is in units of the number of base substitutions per site. Evolutionary analyses were conducted in the MEGA7 program.

Among thirty-six fungal strains isolated from different types of refrigerated cheeses, an MPA producer strain showing high similarity with *P. arizonense* was selected in this study as a new producer strain for MPA. Morphological identification indicated that this species belongs to section *Canescentia*, and molecular analysis of the ITS region confirmed its close relation to *P. arizonense*. Phylogenetic computation according to marker genes proved the grouping of this strain within section *Canescentia***(**Fig. 1_a_), and approximately 98.5–99% similarity was detected with other related strains of *P. arizonense* deposited in the GenBank database. The high similarity of ITS region of our isolate confirmed its morphological identification. Based on both morphological and molecular analyses, the isolated strain was named *P. arizonense* HE-MAwt, and its ITS sequence was submitted to the GenBank database under the accession number MT355884. The wild-type strain was identified using an additional molecular marker *benA* gene and the retrieved sequence was deposited at the GenBank under the accession number ON208859. The phylogenetic analysis according to the *benA* marker gene proved the close relation of our strain to *P. arizonense* strains deposited at GenBank database and confirmed the molecular identification using ITS marker which deposited our isolate within section *Canescentia***(**Fig. 1_b_).

**Fig. 2:**
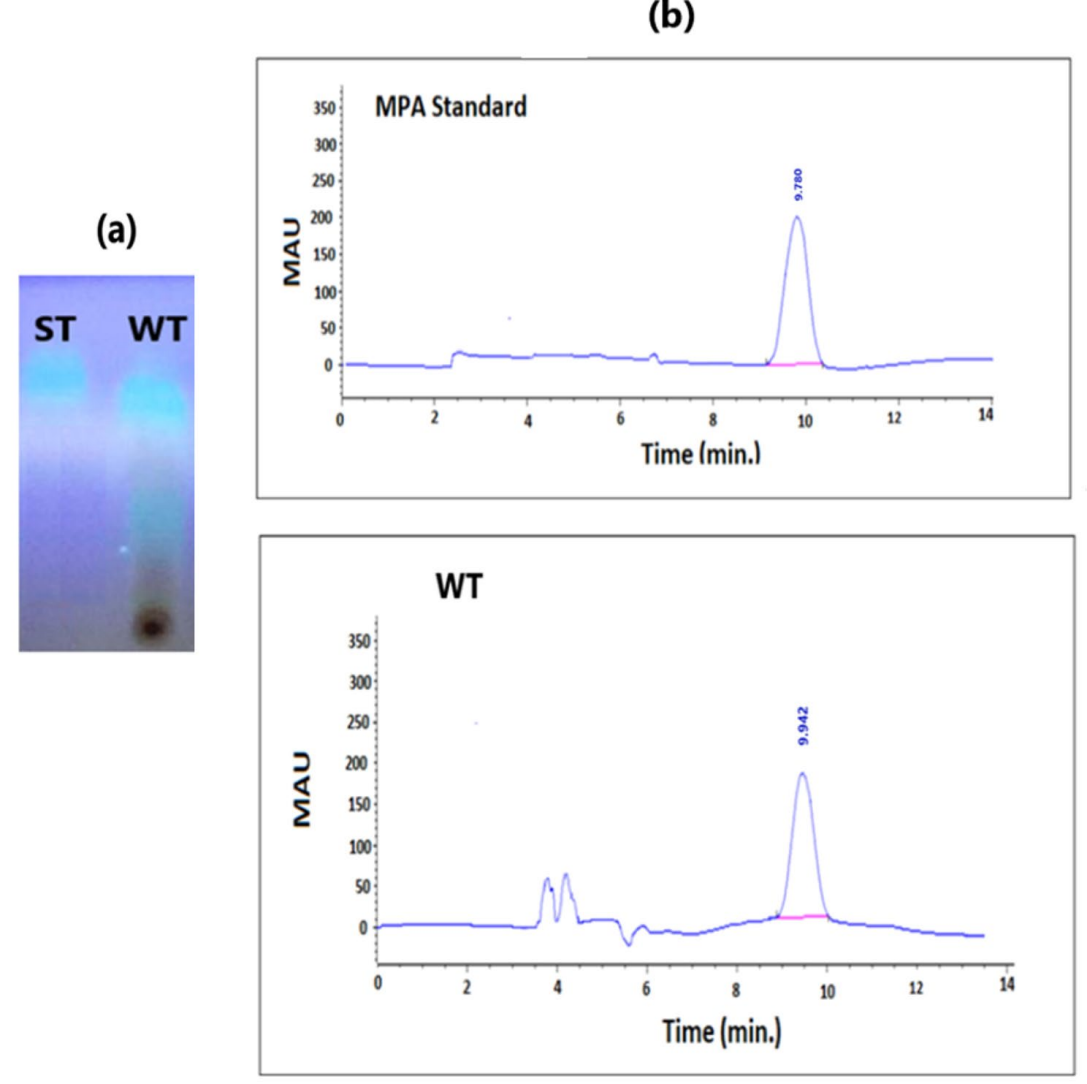
Qualitative and quantitative estimation of MPA produced by wild type *P. arizonense* HE-MAwt (WT) using both TLC (**a**) and (**b**) HPLC techniques, respectively, in presence of standard MPA.

**Fig. 3:**
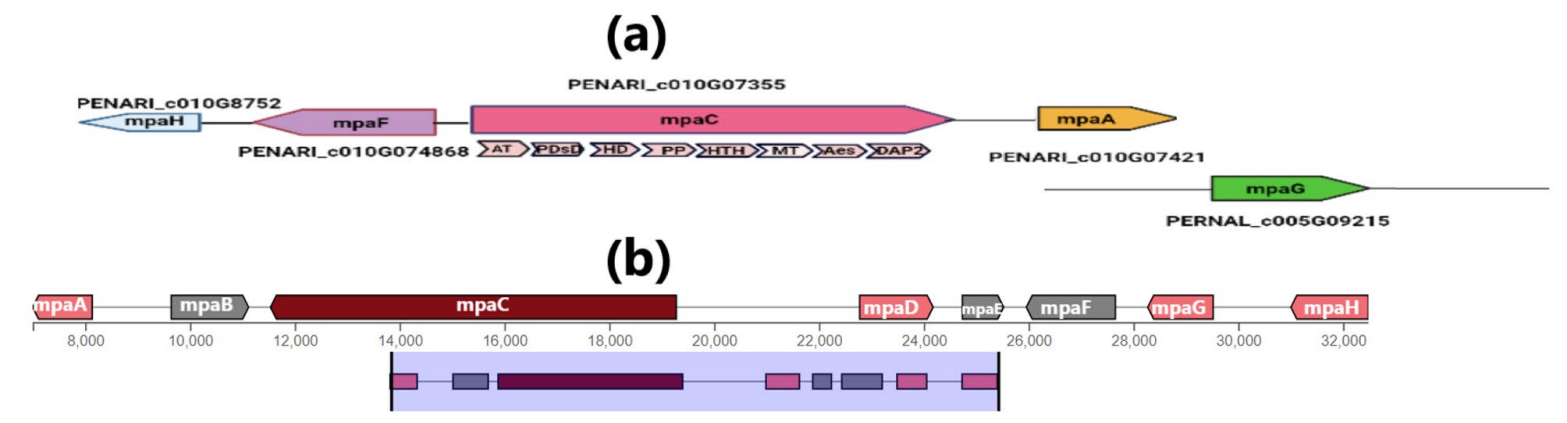
Map of the predicted MPA biosynthesis genes (*mpA, mpC, mpF*, and *mpH*) in *P. arizonense***(a)** and the MPA orthologous gene cluster in *P. brevicompactum* (HQ731031.1) retrieved from MIBiG database **(b).**

**Fig. 4:**
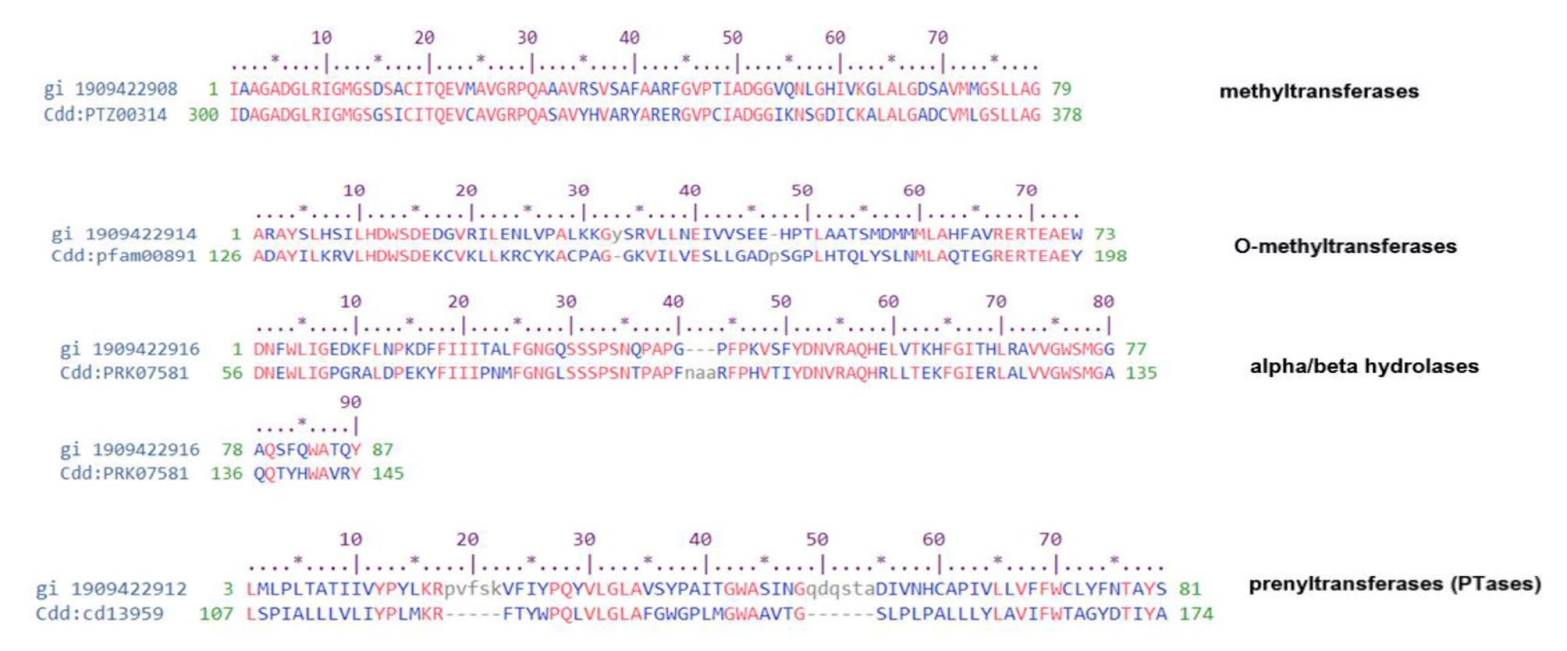
A conserved region of putative protein of MPA gene cluster with respective protein family, gi: our amino acid sequences of MPA gene cluster, cdd: conserved domain of protein family.

The produced compound was initially identified by TLC and then quantified by HPLC analysis compared to the authentic compound. The results presented in Fig. 2_a_ indicated the presence of blue fluorescence spots after exposure of the TLC plate to ammonium vapor in front of the authentic standard compound. The Rf value of the standard solution was equal to the compound separated by our organism. The quantitative determination of MPA produced by wild-type and mutant strains of *P. arizonense* was performed by HPLC analysis, as shown in Fig. 2_b_ and Fig. S. (1), respectively.

### Identification of MPA biosynthetic genes in *P. arizonense*

#### Conventional PCR analysis

PCR analysis showed that the 5 MPA genes, *mpaA, mpaC, mpaF, mpaG*, and *mapH*, gave strong amplification in both wild-type and mutant strains as shown in Fig. S. (2). For more confirmation of the presence of these genes in our isolate and to study the similarity percentage with their orthologous genes in *P. brevicompactum* and their homologous genes in *P. arizonense* strain CBS 141,311, the amplicons resulting from cDNA amplification of the MPA genes (Fig. S. 3) were sequenced in the forward and reverse directions using the designed primers listed in Table S. (1). The obtained sequences of all amplified genes were aligned against the more related genes deposited in the GenBank database using multiple sequence alignment search tools. The blast alignment and bioinformatic analysis of the MPA genes revealed that all retrieved gene sequences showed 96–97% similarity to the related protein-coding genes of *P. arizonense* submitted in the GenBank database and 82–96% similarity to the orthologous genes of MPA biosynthesizing gene clusters of both *P. brevicopmactum* and *P. roquefourti* (Table 1). The retrieved sequences of the amplified genes, *mpaA, mpaC, mpaF, mpaG*, and *mapH* in *P. arizonense* HE-MPwt were registered in the GenBank database under the accession numbers MT786725, MT786724, MT786723, MT786726, and MT797806, respectively (Table S.2). A proposed map of the MPA genes predicted from *P. arizonense* genome and the map of the MPA gene cluster in *P. brevicompactum* (HQ731031.1), retrieved from the MIBiG database (public domain), is represented in Fig. 3. The diagram shows two contige containing five genes in the *P. arizonense* genome that could be responsible for MPA biosynthesis in *P. arizonense.* Bioinformatic analysis of these genes confirmed their close relation to the orthologous genes in both *P. brevicompactum* and *P. roqueforti.* All genes were more related to the homologous genes in *P. arizonense* registered in the GenBank database. The amino acid sequences of the putative proteins also showed high similarity with other orthologous proteins of MPA in both *P. brevicompactum* and *P. roqueforti* (Fig. S. 4), and their conserved regions present in other homologous strains were detected in Fig.(4).

**Table 1:** Percentage of similarity of the retrieved sequences of MPA biosynthesizing genes in *P. arizonense* HEWt1 with both the orthologous MPA biosynthesizing genes and the related protein-coding genes of *P. arizonense* strains submitted in the GenBank database.

MPA gene in *P. arizonense*	similarity with its orthologous gene		similarity with related hypothetical protein coding gene		
	*Penicillium* species	% similarity	*Penicillium* species	Protein locus tag	% similarity
*mpaA*	*P. roqueforti* strain PTX. PR(KU234531.1)	96%	*P. arizonense* (XM_022632298.1)	PENARI_c010G07427	96%
*mpaC*	*P. brevicompactum* (HQ731031.1),	82.05%	*P. arizonense* (XM_022632294.1)	PENARI_c010G07355	93.7%
*mpaF*	*P. brevicompactum* (HQ731031.1),	84.017%	*P. arizonense* (XM_022632233.1)	PENARI_c010G04864	96.25%
*mpaG*	*P. roqueforti* strain PTX.PR.27 (KU234531.1).	82.44%	*P. arizonense* (XM_022629943.1)	PENARI_c005G09215	97.29%
*mpaH*	ND	ND	*P. arizonense* (XM_022632342.1).	PENARI_c010G08752	98.85%

### Overproduction of MPA by mutagenesis of *P*. *arizonense* and mutants’ selection

**Fig. 5:**
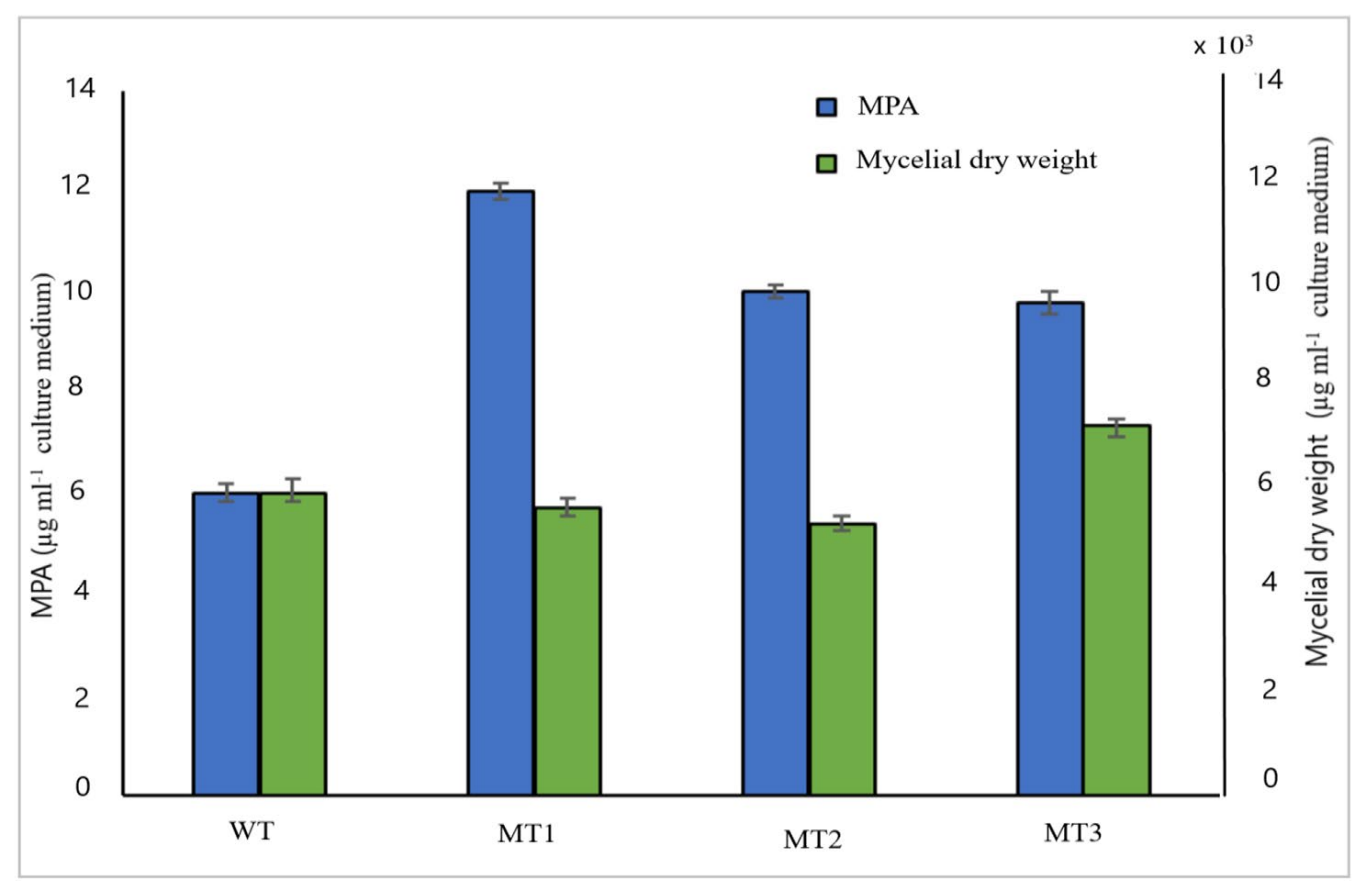
Error bar chart of the change in the mean of mycelial dry weight (µg mL^− 1^ ) and MPA concentrations (µg mL^− 1^) produced by wild type and mutant strains of *P. arizonense* after the first generation. Data are shown as the mean ± SD of triplicate measurements from two independent experiments.

Three mutant strains of *P*. *arizonense*, namely, HE-MPM1, HE-MPM2, and HE-MPM3, were selected as stable overproducer mutants to produce MPA. The initial selection was based on a phenotypic variation of the growing colonies and on the production of MPA in the culture media. The phenotypic characterization of the wild-type and mutant strains proceeded with morphological and microscopic studies after their growth on the PDA culture medium. The phenotypic variation between the mutant and the wild-type strains was summarized in Table S. (3 & 4). It was observed that the mutant strains varied in their growth rate, colony size, and texture of hyphae compared to the wild-type strain as detected in Fig. S. (5 &6) and Table S. (3). There was no change in the color of the metabolite of the wild-type strain, MT1, and MT2 mutant after 5d of incubation on a PDA culture medium, whereas the MT1 and MT2 changed to pale yellow and brownish yellow, respectively after 10 d of incubation. A yellow-colored soluble exudate appeared in the fungal filtrate of MT2 mutant after 5 d of incubation on PD broth and solid culture, which was converted to dark brown after 10 d of incubation. Additionally, small watery droplets, large irregular watery droplets, and yellow condensed irregular exudates appeared on the mycelial growth of wild type, MT1, and MT2 strains, respectively (Fig. S.5). The microscopic characteristics presented in Table S. (4) described that there were no significant variations in the shape and diameter of conidia and phialide of the mutants compared to the wild-type strain. There were no significant differences in the mycelial growth on PD broth culture for wild-type and mutant strains. The mutants HE-MPM1, HE-MPM2, and HE-MPM3 were identified by molecular analysis of the ITS region, and the retrieved sequences were deposited at the GenBank database under the accession numbers MT355885, MT355886, MT355887, respectively. Sequencing analysis showed their high similarity to *P. arizonense* species deposited in the GenBank database and confirmed the previous identification of our wild-type strain. The production of MPA was estimated after each incubation time for four respective generations, as shown in Table S. (5). The results indicated that the selected three mutants maintained the high production of MPA after subculture for 4 generations without significant changes. The MPA amounts produced by MT1, MT2, and MT3 were 11.82 ± 0.174, 9.231 ± 0.178, and 8.79 ± 0.168 µg mL^− 1^, respectively, while the amount produced by the wild-type strain was 5.457 ± 0.174 µg mL^− 1^. These results indicated that MPA production by MT1, MT2, and MT3 was increased by 2.1, 1.7, and 1.6-fold, respectively, compared with the wild-type strain (Fig. 5).

### Optimization of fermentation conditions

**Fig. 6:**
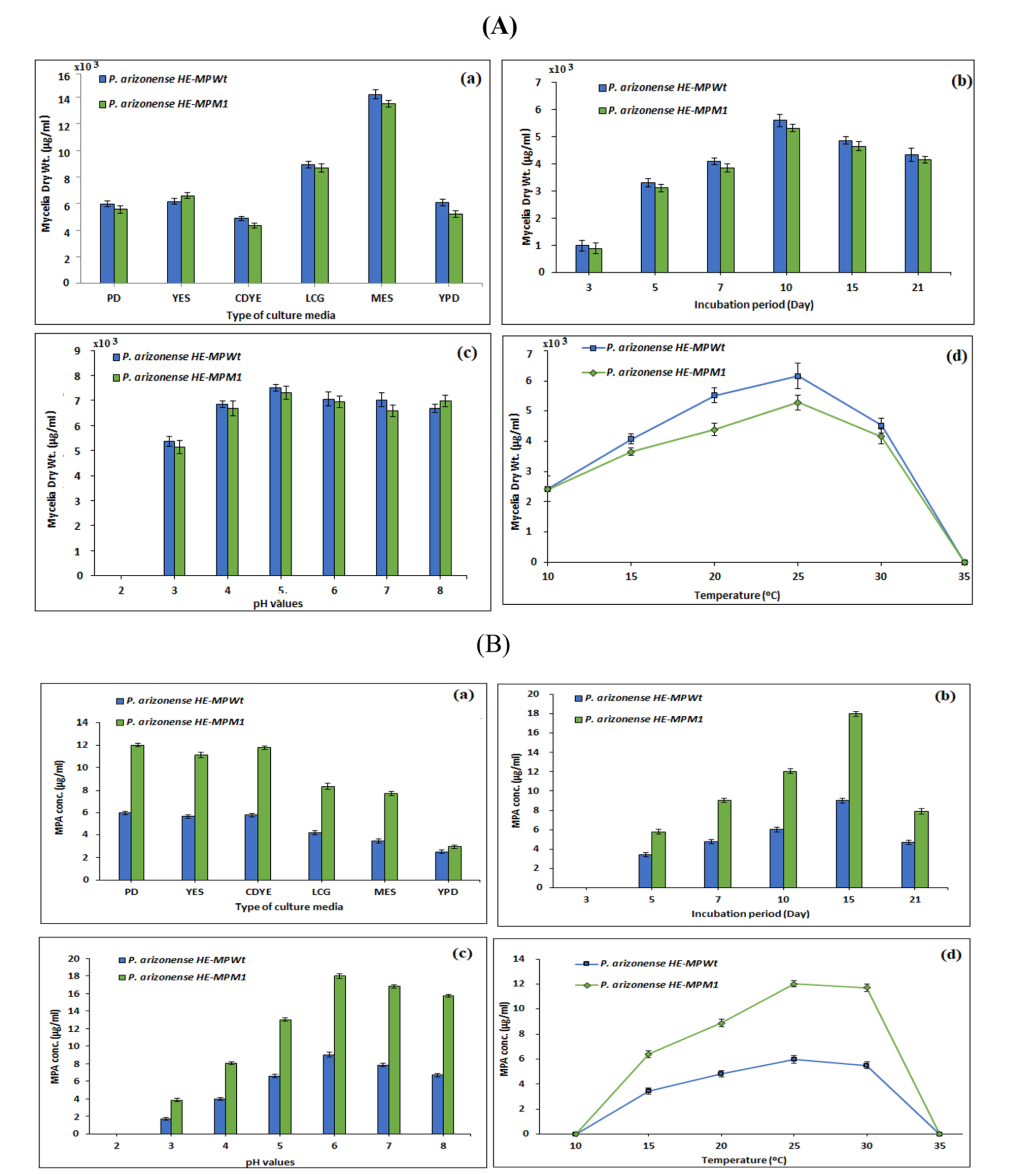
Error bar chart of the change in the mean of (**A**) mycelial dry weights (µg mL^− 1^ culture medium) and (**B**) MPA concentrations (µg mL^− 1^) produced by *P. arizonense* HE-MPWt vs. *P. arizonense* HE-MPM1 grown at different culture media (**a**) and incubated at different incubation times (**b**). The culture media were adjusted to different pH values (**c**) and incubated at different temperatures (**d**). Data are shown as the mean ± SD of triplicate measurements from two independent experiments.

To reach the highest amounts of MPA by our experimental strains, the upstream processing conditions of the production of MPA and fungal growth (Fig. 6 _A,B_) by WT and MT strains of *P. arizonense* were optimized. PD broth medium was the best culture medium for maximum production of MPA by wild-type HE-MPWt and mutant strain HE-MPM1 of *P. arizonense* (5.97449 ± 0.12 and 12.026 ± 0.122 µg mL^− 1^, respectively), whereas MES broth medium was the best culture medium for mycelial dry weight of both wild-type and mutant strains (14.22 ± 0.33 and 13.52.000 ± 0.24 mg mL^− 1^ culture medium, respectively) as shown in Fig. (6_**a**_). It was noticed that the mycelial dry cell weights significantly decreased in the mutant strain compared to the wild-type strain. Optimizing the fermentation conditions was extended to investigate the optimum incubation time for mycelial growth and MPA production. The obtained results from Fig. (6_**b**_**)** revealed that MPA appeared in the culture filtrate of both the HE-MPWt and the HE-MPM1 strains of *P*. *arizonense* after 5 days of incubation (3.441 ± 0.943 and 5.775 ± 0.252 µg mL^− 1^, respectively), and then it was increased by increasing the incubation time until reaching its maximum value (9.032 ± 0.12 and 17.99 ± 0.094 µg mL^− 1^) after 15 days of incubation. On the other hand, the maximum growth of both wild type and mutant strains was observed after 10 d of incubation (Fig. 6_**b**_). The growth and the production of MPA by the two experimental strains were also greatly affected by the initial pH value of the culture medium (Fig. 6_**c**_). The results showed that MPA production by *P. arizonense* HE-MPWt and *P*. *arizonense* HE-MPM1 increased gradually by increasing the pH value of the culture medium, reaching maximum values (9.0 ± 0.27 and 17.98 ± 0.285 µg mL^− 1^, respectively) at pH 6 after 15 days of incubation at 25 °C. It was noticed that the ranges of pH values suitable for mycelial growth ranged from 5 to 8 while the maximum growth of both wild type and mutant strains was observed at pH value 5 (7.50 ± 1.20 and 7.31 ± 1.57 mg mL^− 1^ culture medium, respectively). After incubation of both the wild-type and mutant strains at 10, 15, 20, 25, 30, and 35 °C for 10 days, the optimum incubation temperature for optimum production of MPA by both *P. arizonense* HE-MPWt and *P. arizonense* HE-MPM1 strains (5.974 ± 0.31 and 12.1 ± 0.2404 µg mL^− 1^, respectively) and for maximum mycelial growth (6.16 ± 0.42and 5.28 ± 0.29 mg mL^− 1^ culture medium, respectively) was recorded at 25 °C **(**Fig. 6_**d**_**)**. Although well growth of both mutants and wild type was observed at 10 °C, all strains failed to produce MPA at this temperature. These results indicate that the growth of all experimental strains on PD broth adjusted to pH 6 and incubated at 25 °C for 15 d were favorable for MPA production. In general, there were significant differences in MPA production by the wild-type and all mutant strains under all tested environmental conditions according to independent samples t-tests (P ≤ 0.05). Additionally, there was no relation between the mycelial growth and the MPA production in most of the tested conditions. The optimal environmental conditions of the three selected mutants were identical in all experiments. Therefore, the highest producer mutant, MT1, was represented in this study as a model mutant in comparison with the wild-type strain.

### Quantitative real-time reverse transcription (qRT-PCR) analysis of MPA biosynthetic genes in *P. arizonense*

**Fig. 7:**
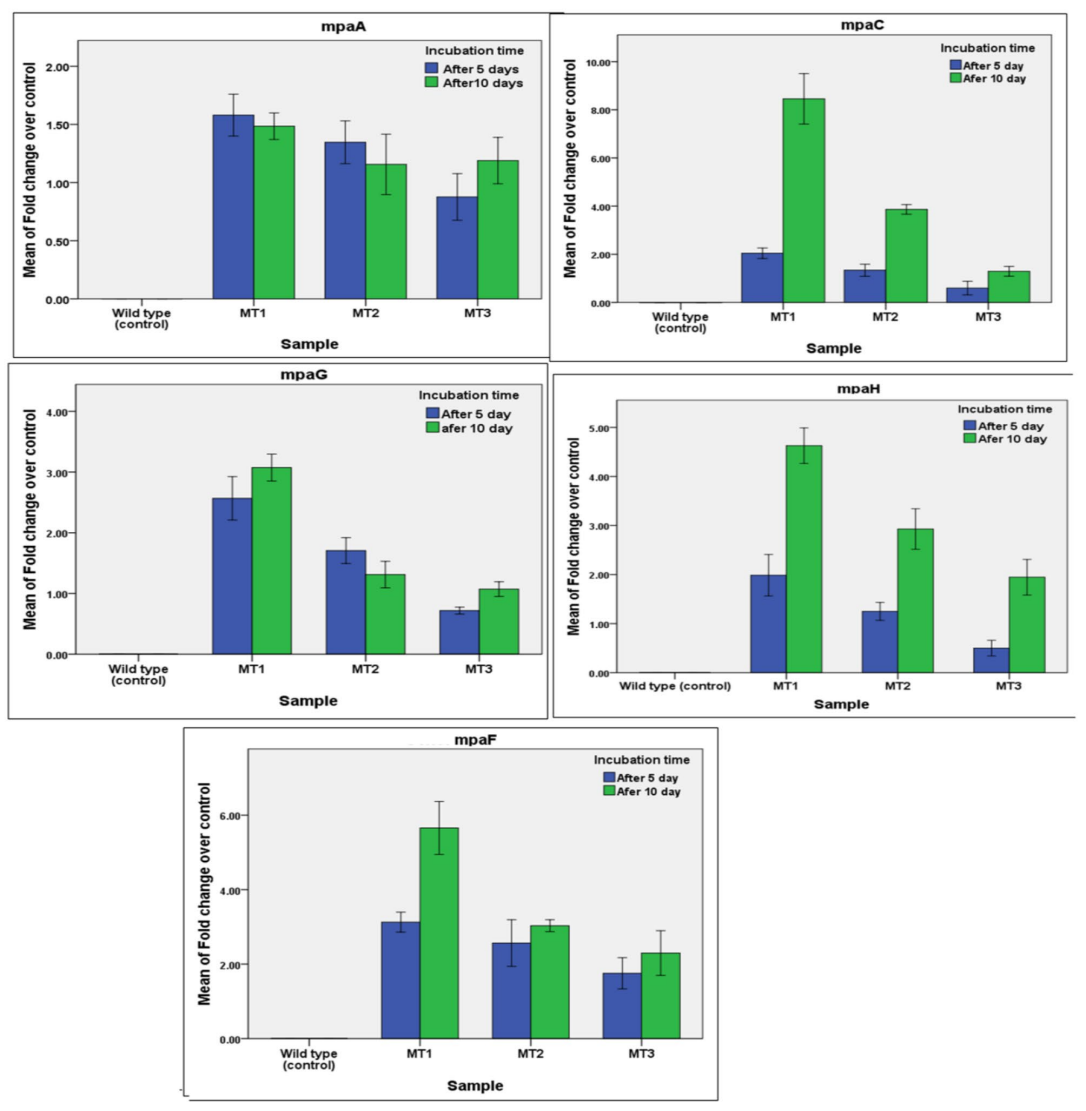
Gene expression analysis of MPA gene cluster after incubation of overproducer mutant *P. arizonense* HE-MPM1 for 5 and 10 d on MPA producer culture medium compared with the expression of MPA gene cluster in the wild type *P. arizonense* HE-MAwt incubated at the same environmental conditions. Data are shown as the mean ± SD of triplicate measurements from two independent experiments.

The qRT-PCR results were analyzed with iQ 5 optical system software (Bio-Rad) using the PCR baseline subtracted mode. The threshold cycle was calculated for all samples, and the amplification efficiency for each gene amplified from *P. arizonense* (WT and MT strains) was determined. The threshold cycle of all genes, in addition to the housekeeping gene (*β -actin* gene), of *P. arizonense* (WT strain HE-MPwt and mutant strains MT1, MT2, and MT3) after 5 d and 10 d of incubation time are shown in Table S. (6) and Fig. (7). The wild-type strain was used as endogenous control, and the *β-actin* gene was used for normalization as control of constant expression. Table S. (6) shows the expression of the MPA genes, *mpaA, mpaC, mpaF, mpaG, and mpaH* in MPA over producer mutant strains after 5 and 10 days of incubation. The obtained results recorded a significant increase in the expression of all analyzed mycophenolic acid genes in the three tested mycophenolic acids producer mutants over the wild-type strain of *P. arizonense*. It was observed that the transcription value of these genes in mutant strain MT3 was the highest compared to the other mutant strains. The transcription values of *mpaA, mpaC, mpaF, mpaG*, and *mpaH* in mutant strain MT1 were1.6, 2.0, 3.1, 2.6 and 1.99 after 5 days, and 1.5, 8.5, 5.7, 3.1, and 4.6-fold higher than those in the wild-type strain after 10 d of incubation, respectively. It was observed that the gene expression of *mpaC, mpaF, mpaH*, and *mpaG* was significantly increased according to the incubation time, and these increments corresponded to the production of MPA, where its amount doubled after 10 d of incubation. The expression values of *mpaC* and *mpaF* were significantly higher than those of the other genes according to t-test analysis, where the *p*-value was lower than 0.05. The expression values of *mpaC* and *mpaF* were the highest in all mutants, followed by *mpaH* and *mpaG*, whereas the expression value of *mpaA* was the lowest at all. It was also noticed that the expression of the MPA genes in the mutant strain MT1 was higher than that of MT2 and MT3 after 5 and 10 days of incubation. The expression of the identified genes was correlated with the production of MPA, where the mutant strain MT1 was the highest producer strain for MPA and the production of MPA significantly increased after 10 days of incubation. The recorded results confirmed the potential roles of the five genes *mpaA*, *mpaC*, *mpaG*, *mpaF*, and *mpaH* in the biosynthesis process of MPA in *P. arizonense*.

## Discussion

Mycophenolic acid is a fungal secondary metabolite with a variety of pharmaceutical activities and is widely applied in clinical practices. For its vital importance in the biomedical field, in this study we aimed to isolate a new MPA producer species from the genus *Penicillium* and to improve its production. *Penicillium* is a vital industrial unit for the biosynthesis of many chemically and structurally diverse secondary metabolites, including significant pharmaceutical compounds [3]. These compounds include penicillin, roquefortine, chrysogine, and fungisporin which represent the starting point for the investigation of highly efficient antibiotics, a milestone in therapeutic medicine [45]. The most principal scientific discovery of the 20th century was Penicillin antibiotics which were among the first drugs to be effective against several diseases [46]. MPA is one of the most important secondary metabolites, which was discovered in 1893 and has been identified as an essential bioactive substance with immunosuppressive, antibacterial, antifungal, antiviral, antitumor, and various activities. It has been produced by many *Penicillium* species including *P. brevicompactum* (also known as *P. stoloniferum*), *P. paxilli, P. olivicolor, P. canescens* (also known as *P. raciborskii*), *P. roqueforti, and P. viridicatum* [47, 48]. In this study, we isolated a new antibiotic-producer strain of *P. arizonense* from refrigerated Mozzarella cheese, and it was found to be a strong producer of MPA when it was grown in submerged PD broth culture. Large numbers of secondary metabolites, such as tryptoquivalines, pyripyropenes, austalides, fumagillin, pseurotin A, xanthoepocin, and curvulinic acid, were investigated in the cell extract of *P. arizonense* [3]. The morphological identification and marker gene analysis of rRNA and *benA* confirmed the highest similarity of our isolate with *P. arizonense* CBS141311. Because many of the preferable compounds are naturally produced in considerable amounts, fungi have a great potential as specific hosts for the biosynthesis of small molecules. The broad benefit in fungi has led to the hole sequencing of numerous fungal genomes. This number is expected to a dramatically increase in the upcoming years [49]. Information concerning the molecular basis of MPA biosynthesis in *P. arizonense* would be very helpful for both the potential production of MPA and the control of its contamination in food products; however, there is no previous information about the biosynthetic pathway. In this study, two contigs containing five orthologous genes of MPA gene clusters were identified in the *P. arizonense* genome, namely, *mpaA, mpC, mpaF*, *mpaG*, and *mpaH*. The genes *mpaA*, which encodes a putative prenyltransferase; *mpaC*, which encodes a polyketide synthase; *mpaDE*, which encodes a natural fusion of a cytochrome P450 domain and a hydrolase domain; *mpaF*, which encodes a protein with high similarity to inosine-5´-monophosphate dehydrogenase (IMPDH); *mpaG*, which encodes an O-methyltransferase; and *mpaH*, which encodes an oxidative cleavage enzyme, were identified as gene clusters for MPA biosynthesis in the *P. brevicompactum* genome. The *mpaC* gene is responsible for the biosynthesis of 5-methylorsellinic acid (5-MOA), which is the initial step in MPA synthesis. The following conversion of 5-MOA to 4,6-dihydroxy-2-(hydroxymethyl)-3-methylbenzoic acid and 5,7-dihydroxy-4-methyl phthalide (DHMP) was performed by the enzyme *mpaDE*. The final step in the biosynthesis process is completed by *mpaG* (the putative O-methyl transferase), which stimulates the methylation of dimethyl mycophenolic acid (DMMPA) to form MPA. The strong amplification of these genes from the *P. arizonense* genome indicates their participation in the biosynthesis of MPA. In silico study, no contig containing the ORFs encoding similar proteins to *mpaB, mpaE*, and *mpaD* were identified in the genome of *P. arizonense*. This result indicated that there are no homologous sequences of the three unidentified genes, *mpaB, mpaE, and mpaD*, with the related orthologous genes of both *P. brevicompactum* and *P. roqueforti*. To predict these genes, additional studies are required for the identification of the putative proteins encoded by these genes. The gene cluster encoding MPA was recently investigated in both *P. brevicompactum* [6] and *P. roqueforti* [22, 50]. *P. arizonense* is defined as one of a group that belongs to the *Penicillium* section *Canescentia* and contains a large number of genes participating in lipid metabolism and a secondary metabolite production. Sequencing analysis of the *P. arizonense* genome revealed that it was assembled into 33.7 Mb containing 12,502 predicted genes [3]. Additionally, 62 putative biosynthetic gene clusters involved in secondary metabolite biosynthesis were identified in its genome. Up till now, the putative genes that participated in the *P. arizonense* genome have not been completely annotated, and their phenotypic action is still under investigation. It was previously investigated whether *P. arizonense* contains gene clusters responsible for austalide (meroterpenoid) production. Mycophenolic acid is an important compound related to meroterpenoids that is composed of a terpene-derived side chain and an acetate-derived phthalide nucleus [51]. This phthalide structure is also present in the austalides, so it is likely that austalides and mycophenolic acid have identical biosynthetic gene clusters. Orthologs to *mpaC, mpaD*, and *mpaA* genes evolving in the MPA biosynthesis cluster of *P. brevicompactum* were found in the *P. arizonense* genome [3]. This report agrees with our investigation, where a homologous partial sequence of 5 genes with locus tags, PENARI_c010G07427, PENARI_c010G07355, PENARI_c010G04864, PENARI_c005G09215, and PENARI_c010G08752, were annotated in the *P. arizonense* genome, and they were found to be orthologs to the MPA gene cluster *mpaA, mpaC, mpaF, mpaG, and mpaH*, respectively, in *P. brevicompactum*. The use of mutations to improve several microorganisms for the overproduction of industrial products has been applied for over 50 years and is still accepted as a valuable agent for the improvement of different microbial strains. The random mutagenesis against the wild type of fungal strains leads to a series of mutants, some of them change the production of target secondary metabolite. Most improvements lead to a decrease or lack of production change, but some mutants may show an increase in the production of secondary metabolite. Through the exposure of wild-type *P. arizonense* to random mutagenesis by gamma irradiation, potential producer mutants for MPA were obtained. We succeeded in isolating three stable mutants of *P. arizonense* that had the ability to produce approximately double the amount of MPA compared with the wild-type strain. This result indicates the technological modification of our isolate to be more productive for MPA. Such a mutagenic strain could minimize the cost of the manufacturing processes and enhance the productivity of MPA. The improvement of MPA production was previously investigated by exposing *P. brevicompactum* to 250 Gy of gamma radiation, which led to an increase in MPA production productivity by 25% [38]. Gamma rays may cause various mutations in the gene cluster regulating several secondary metabolites, leading to an increase or decrease in their production [52]. These metabolites are regulated by a network of master regulators. One such regulator is the Velvet protein complex which is mainly conserved as the regulator of the fungal evaluation and secondary metabolism [53]. A key regulator of the biosynthesis of secondary metabolites is the LAEA protein that interacts with other components of the velvet complex. The overproduction of MPA may also result from the modification of different processes such as inhibition or secretion of other secondary metabolites in the mutant strains. The variation in the colored pigments of the mutant’s metabolite indicated the production of other secondary metabolites compared to the wild-type strain. This result indicated that the mutated programs affect other genes such as *lea* and *velvet* genes which control secondary metabolites production and this likely has impacted the expression of several secondary metabolites encoding genes [54]. Because *P. arizonense* is considered a new producer strain for MPA, the eco-physiological parameters for its production must be optimized to achieve maximum productivity. The incubation temperature and time were effective factors in the production activity of MPA by both wild-type and mutant strains. Generally, like other secondary metabolites, the production of MPA was noticed to be increased during the stationary phase of fungal growth. MPA is a secondary metabolite, so its production was affected by the time of incubation, where the elongation of incubation time to 10 and 15 days enhanced the productivity of our experimental strains. Similarly, **Vinokurova et al.** [55] reported that MPA synthesis was dramatically concentrated during the stationary phase of growth after 10 days of incubation. This investigation agrees with our results, where the mycelial growth of all mutant and wild-type strains did not correlate with MPA production. Additionally, the initial pH value and the incubation temperature were critical factors affecting the production of MPA by *P. arizonense*. The highest production of MPA was observed when all fungal strains were grown on PD broth adjusted to initial pH 6 and incubated at 25 °C. Accompanied by our results, **Patel et al.** [56] recorded that the optimum culture conditions for maximum production of MPA were detected after incubation of *P. brevicompactum* for 12 d at 25 °C using pH 5 as an initial value. Additionally, **Wu et al.**. [57] reported that MPA production was 58.1% higher under optimum fermentation conditions than that initial fermentation condition without optimizations. Overproduction of MPA in mutated strains was confirmed with expression analysis of the main predicted MPA biosynthesizing genes. Expression analysis of these genes in all MPA over producer mutants showed a highly significant fold increase over the wild type after incubation times of 5 and 10 days. Additionally, the expression values of all genes were higher after 10 d than after 5 d of incubation. This result agrees with the physiological study of MPA production by mutant and wild-type strains of *P. arizonense*, where the amount of MPA was significantly higher in all mutant strains compared to wild-type and increased after 10 d of incubation compared to 5 d. The obtained results indicated a positive relationship between the transcription value of the MPA genes, *mpaA, mpaC, mpaF, mpaG, and mpaH*, and the production of MPA under different environmental conditions. Additionally, these results confirmed the vital role of the annotated genes in the biosynthesis pathway, which was considered an indication of the capability of the *P. arizonense* HEwt strain to produce MPA in submerged culture. The significant similarity between the five sequence amplicons with their related putative protein-encoding genes of *P. arizonense* CBS141311, deposited in the GenBank database, is considered strong evidence for the correct identification of our strain. This is the first report concerning the overproduction of MPA by *P. arizonense*, in addition to the expression analysis of the orthologous MPA genes and their correlation with phenotypic analysis. This investigation could be a new platform for the industrial biosynthesis of this vital compound using a new producer species of *Penicillium* and considered a new annotation of five MPA genes shared in MPA biosynthesis in the *P. arizonense* genome.

## Conclusion

We concluded from this study that the *Penicillium* group contains various species that have the capability to produce several important secondary metabolites. A new local strain *of P. arisonense* was isolated from refrigerated cheese and identified *as P. arizonense* HE-wt. This strain showed high productivity for MPA during its growth in PD broth. The production of MPA was improved by mutagenesis of the wild type and isolation of three stronger stable mutants for MPA production. The optimized conditions for maximum production of MPA by both mutant and wild type revealed that the growth of all strains on PD broth adjusted to pH 6 and incubated at 25 °C for 15 d were optimum for MPA production. The molecular basis for MPA production in *P. arizonense* was also investigated in our research. Through bioinformatic analysis and partial sequencing analysis of the MPA ortholog genes, we confirmed the presence of five open reading frames of locus tags PENARI_c010G07427, PENARI_c010G07355, PENARI_c010G04864, PENARI_c005G09215, and PENARI_c010G08752, that were orthologous to the MPA gene cluster *mpaA, mpaC, mpaF, mpaG, and mpaH* in *P. arizonense* HE genome. The phenotypic and genotypic analyses showed their importance in the biosynthesis of MPA by *P. arizonense* and suggested that three of them, namely, *mpaC, mpaF, and mpaG*, have an intensive role in the biosynthesis process. This is the first report regarding the isolation of a new MPA producer strain belonging to section *Canescentia* and the improvement of this production by mutagenesis of the wildtype strain by Gama radiation in addition to the expression analysis of the biosynthesizing orthologous genes in *P. arizonense* genome.

## Electronic supplementary material

Below is the link to the electronic supplementary material.

Supplementary Material 1

Supplementary Material 2

## Data Availability

The authors declare that all the data supporting the work are available within the paper and its supplementary information.

## Code Availability

All programs and software used in this study are freely available and are referred to in the Method section.
