# Supplementary material for: Titer improvement of mycophenolic acid in the novel producer strain Penicillium arizonense and expression analysis of its biosynthetic genes
Source: BMC Microbiol. 2023 May 17;23:135. doi: 10.1186/s12866-023-02884-z (PMC10189230; doi:10.1186/s12866-023-02884-z)
Supplement: Supplementary file 1 — Supplementary Material 1 [file 12866_2023_2884_MOESM1_ESM.pdf]

Table S. (1) Primer sequences of the MPA gene cluster designed for conventional and qRT–PCR analyses, and primers sequences used in molecular identification.

Target genes	Primer name	Primer sequences<br>5′ to 3′	Amplicon length (bp)	Tm	Amplification	References
mpaA	mpaA	F-TAGGGTGGCTCGGACAAAATG<br>R-TCTTGCCCATTGATCGAAGC	288	60.3<br>58.8	+ve	Current study
mpaC	mpaC	F-TGACACGCAGAGCATTTGAC<br>R-TCGACCAATTGTGTCCATGC	283	59.1<br>59.8	+ve	Current study
mpaF	mpaF	F-TTGATACCCCTGTCACCAAGC<br>R-ACTTGGCCTTGAGCTCCTTG	258	60<br>60.3	+ve	Current study
mpaG	mpaG	F-CCCATGACATCTTCACAACGC<br>R-ATCTCCAATCGGCCTCTGTTC	258	59.9<br>59.9	+ve	Current study
mpaH	mpaH	F-TGGTTGATCGGCGAAGACAAG<br>R-TACTGAGTCGCCCATTGGAAAG	251	60.9<br>60.4	+ve	Current study
β-actin	ACT 512<br>ACT 783	F-ATG TGC AAG GCC GGT TTC GC<br>R (5' TAC GAG TCC TTC TGG CCC AT	300	61<br>58	+ve	Carbone and Kohn [42]
Internal Transcribed Spacer (ITS)	ITS1<br>ITS4	F-TCCGTAGGTGAACCTGCGG<br>R-TCCTCCGCTTATTGATATGC	620	65<br>58	+ve	White et al. [32]
β-tubulin (*benA*)	Bt2a<br>Bt2b	F-GGTAACCAAATCGGTGCTGCTTT R-ACCCTCAGTGTAGTGACCCTTGGC	440	55<br>57	+ve	Visagie et al. [1]

Table S. (2) Accession numbers and full annotation of the MPA biosynthesizing genes predicted from the *P. arizonense* genome. Annotation was acquired from the NCBI database.

**Accession number**	**Standard name**	**Locus tag in *P. arizonense***	**Gene description**
**MT786725**	*mpaA*	PENARI_c010G07427	Polyprenyl transferase; part of the gene cluster that mediates the biosynthesis of mycophenolic acid
**MT786724**	*mpaC*	PENARI_c010G07355	polyketide synthase<br>catalyzing the synthesis of 5-methyl orsellinic acid, first step of mycophenolic acid biosynthesis"
**MT786723**	*mpaF*	PENARI_c010G04864	Inosine-5'-monophosphate dehydrogenase

**MT786726**	*mpaG*	PENARI_c005G09215	S-adenosylmethionine-dependent methyltransferase (SAM or AdoMet-MTase), class I; AdoMet-MTases are enzymes that use S-adenosyl-L-methionine (SAM or AdoMet) as a substrate for methyltransfer, creating the product S-adenosyl-L-homocysteine (AdoHcy)
**MT797806**	*mpaH*	PENARI_c010G08752	alpha/beta hydrolases

Table S. (3) culture characterization of wild type and mutant strains of *P. arizonense* HEM-MP growing on PD broth and solid culture medium after different incubation time.

**Strains**	**Colony diameter on plate culture (mm)**			**Mycelial growth (mg ml^{-1}) culture medium**			**Color of metabolite**	**Colony texture**	**sporulation**	**Exudate**
	**incubation time (day)**									
	**5**	**7**	**10**	**5**	**7**	**10**				
**Wild type**	8.1±1.00	25.2±2.74	33.8±1.93	3.29±0.314	4.09±0.23	5.58±0.29	Pale yellow	woolly	Condensed-Pale green	Little watery droplet
**MT1**	5.7±1.07	21.3±1.58	29.9±3.04	2.59±0.53	3.45±0.23	4.89±0.29	Soluble brownish pigment appeared after 10 d	Fine smooth	Moderate-greenish white	Observed pale yellow droplet
**MT2**	5.5±1.72	20.1±2.06	26.7±1.28	2.47±0.33	3.07±0.23	4.25±0.31	Dark brown after 10 d	cottony	Moderate dark lemon green	Large dark yellow exudate
**MT3**	4.3±1.75	19.4±2.31	26.4±1.93	1.99±0.20	3.61±0.23	4.34±0.32	Little darkness about culture medium	Rough irregular with smooth batch inside the mat	Sparse- gray	No exudate

Table S (4) microscopic characterization of wild type and mutant strains of *P. arizonense* HEM-MP

**Strain**	**Conidia length (µm)**	**Conidia width (µm)**	**shape of conidia**	**Embellishment of conidia**	**Phialide shape**
**Wild type**	2.5	2.2	Globose, sub-globose	Smooth	Flask shape
**MT1**	2.6	2.3	Globose	Smooth	ampulliform shape with contraction
**MT2**	2.5	2.3	Globose, subglobose	Smooth	Flask shape
**MT3**	2.5	2.1	Ellipsoid- Globose	finely roughened wall	Flask shape

Table S. (5) Stability study of the mutant strains of *P. arizonense* grown on PD broth medium through four sequencing generations showing MPA amounts (µg ml^{-1} culture medium) and mycelial dry weight (mg ml^{-1})

	2nd generation		3rd generation		4th generation	
	MPA concentration	Mycelial dry weight	MPA	Mycelial dry weight	MPA	Mycelial dry weight
Wild type	5.971±0.184[a]	5.84±0.19[a]	5.492±0.193 [a]	5.57±0.36 a	5.457±0.174 [a]	5.48±0.348 [a]
MT1	12.019±0.157 [a,b]	5.547±0.34 a	11.509±0.174 [a,b]	5.28±0.17 a	11.82±0.174 [a,b]	5.04±0.214 [a]
MT2	9.81 ±0.163[a,b]	5.36±0.22 a,b	9.416±0.189 [a,b]	5.05±0.29 a,b	9.231±0.178[a,b]	4.98±0.44 [a,b]
MT3	9.573±0.158 [a,b]	7.18±0.16 a,b	8.972±0.174 [a,b]	6.90±0.45 a,b	8.79±0.168 [a,b]	6.90±0.39 [a,b]

Data represented as the mean ± standard deviation (SD). Calculated mean is for triplicate measurements.
[a]: Statistically significant at $P \leq 0.05$ according to one-way ANOVA.
[b]: Statistically significant at $P \leq 0.05$ when compared pairwise with the 1st variable (Control) by Tukey's test.
1.58, 2.0420, 3.1383, 2.5669, 1.9862

Table S. (6): Threshold cycles (CT) and fold change to control of mycophenolic acid gene cluster *mpaA, mpaC, mpaF, mpaG* and mpaH genes and housekeeping gene (*B. actin*) amplified from wild (HE-MAwt) and mutants (MT1, MT2 and MT3) of *penicillium arizonense* genome, after 5 and 10 days of incubation. The expression of each gene was defined as the fold change normalized to the reference gene, *B. actin*, and relative to the wild type as a control sample.

Genes	Sample	After 5 days of Incubation periods			After 10 days of incubation			p value[a]
		CT value of *B. actin* gene	CT value of target gene	Fold change over control	CT value of *B. actin* gene	CT value of target gene	Fold change over control	
mpaA	Wild type (control)	21.24±0.98	23.00±3.50	-	20.29±1.01	0.00±0.00	-	-
	MT1	20.56±1.23	21.66±0.37	1.58±0.09[c]	19.41±1	21.70±0.69	1.4845±0.16 [c]	**0.037 [a]**
	MT2	20.08±1.17	21.38±1.23	1.3755±0.35 [c]	20.55±1.13	21.65±1.23	1.567±0.17 [c]	**0.013 [a]**
	MT3	20.17±1.0	22.12±0.90	0.8766±0.1 [c]	20.62±0.71	20.20±0.72	1.1892±0.1 [c]	**0.033[a]**
p value[b]				**<0.001[b]**			**<0.001[b]**	

**mpaC**	**Wild type (control)**	21.24±1.78	22.60±0.92	-	20.29±1.03	24.21±0.88	-	-
	**MT1**	20.56±1.02	20.89 ±1.16	2.0420±0.109 [c]	20.55±2.30	22.85±1.88	8.4561±1.02 [c]	**0.003 [a]**
	**MT2**	20.08±0.16	21.02±1.0	1.3379±0.13 [c]	19.41±1.0	22.94±0.99	3.8637±0.10 [c]	**0.003[a]**
	**MT3**	20.17±0.92	22.28 ±1.77	0.5946±0.14 [c]	20.62±0.71	24.44±0.79	1.2924±0.10	**0.001[a]**
**p value[b]**				**<0.001[b]**			**<0.001[b]**	
**mpaF**	**Wild type (control)**	21.24±0.92	23.92±0.77	-	20.29±1.11	24.18±0.97	-	-
	**MT1**	20.56±1.67	21.59±2.14	3.1383±0.13 [c]	20.55±1.77	21.94±1.93	5.6569±0.87 [c]	**0. 012 [a]**
	**MT2**	20.08±1.11	21.40±0.80	2.5669±0.31 [c]	19.41±0.59	21.70±1.11	3.0314±0.08 [c]	0.177
	**MT3**	20.17±1.08	22.04±1.01	1.7532±0.79 [c]	20.62±0.82	23.31±1.3	2.2974±0.3 [c]	0.206
**p value[b]**				**<0.001[b]**			**<0.001[b]**	
**mpaG**	**Wild type (control)**	21.24±0.81	22.89±1.96	-	20.29±1.0	24.21±1.19	-	-
	**MT1**	20.56±0.82	20.85±1.89	2.5669±0.62 [c]	20.55±0.58	22.85±1.88	3.0738±0.11 [c]	0.094
	**MT2**	20.08±0.99	20.96±0.95	1.7053±0.11[c]	19.41±0.59	22.94±0.92	1.3104±0.41 [c]	0.087
	**MT3**	20.17±0.88	22.30±0.40	0.7170±0.19 [c]	20.62±0.32	24.44±0.58	1.0718±0.06 [c]	**0.021 [a]**
**p value[b]**				**<0.001[b]**			**<0.001[b]**	
**mpaH**	**Wild type (control)**	21.24±0.82	22.40± 0.7	-	20.29±0.63	24.53±0.79	-	-
	**MT1**	20.56±0.7	20.73±0.83	1.9862±0.21 [c]	20.55±1.26	22.58±0.71	4.6268±0.18 [c]	**<0.001[a]**
	**MT2**	20.08±1.02	20.92±0.94	1.2483±0.09 [c]	19.41±0.62	22.10±1.10	2.9282±0.21 [c]	**0 .010 [a]**
	**MT3**	20.17±0.99	22.33±0.69	0.50±0.08 [c]	20.62±.68	23.90±0.24	1.9453±0.18 [c]	**0.002[a]**

**p value[b]**	**<0.001[b]**		**<0.001[b]**	

a: Statistically significant at $P < 0.05$ according to paired t test.
b: Statistically significant at $P < 0.05$ according to one-way ANOVA.
c: Statistically significant at $P < 0.0125$ according to post hoc adjusted by Bonferroni's corrections for pairwise comparison using the wild type (as a control).
