# Supplementary material for: Titer improvement of mycophenolic acid in the novel producer strain Penicillium arizonense and expression analysis of its biosynthetic genes
Source: BMC Microbiol. 2023 May 17;23:135. doi: 10.1186/s12866-023-02884-z (PMC10189230; doi:10.1186/s12866-023-02884-z)
Supplement: Supplementary file 2 — Supplementary Material 2 [file 12866_2023_2884_MOESM2_ESM.pdf]

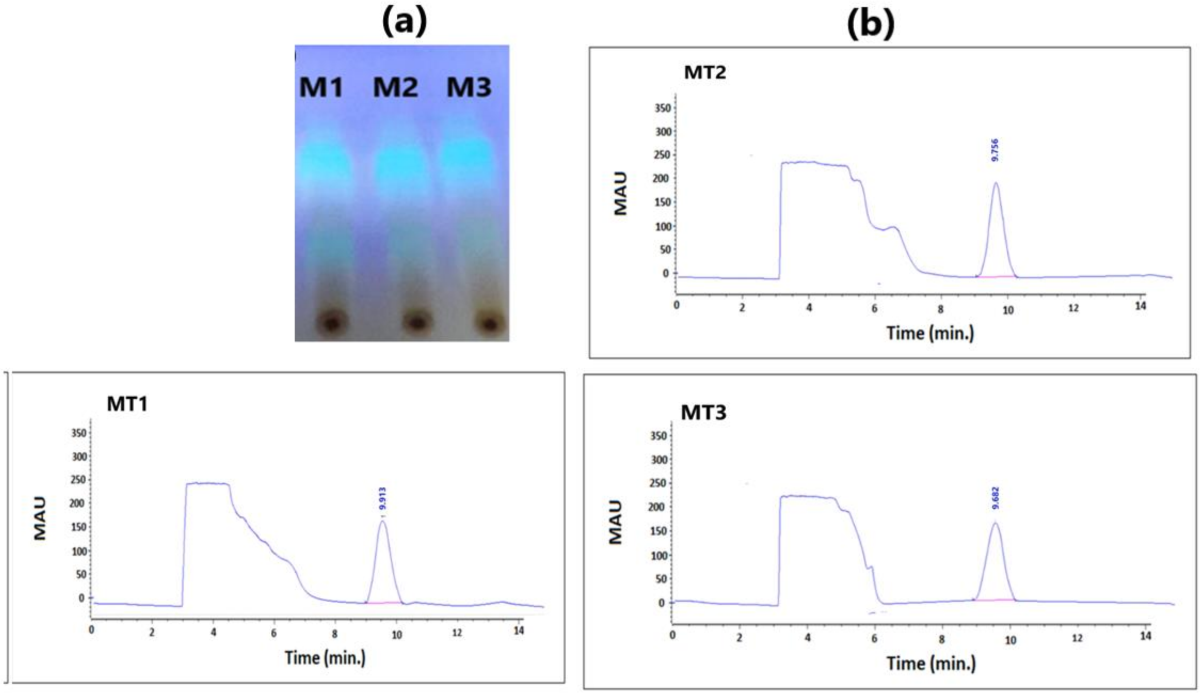

Fig. S. (1) Qualitative and quantitative analyses of MPA produced by mutant strains *P. arizonense* HE-MPM1 (MT1), HE-MPM2 (MT2), and HE-MPM1 (MT3) using both TLC (a) and HPLC (b) techniques, respectively.

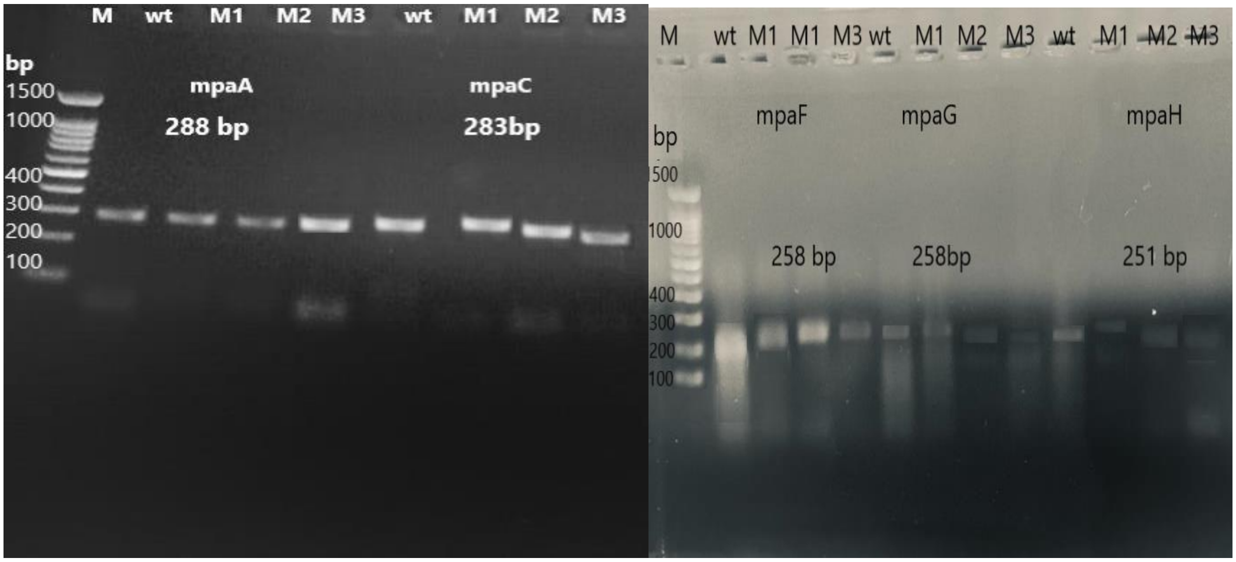

Fig. S. (2) Agarose gel electrophoresis of MPA gene cluster (*mpaA, mpaC, mpaF, mpaG* and *mpaH*) amplified from genomic DNA of wild type HE-MTwt (wt), and MPA overproducer mutant strains HE-MPM1. (M1), MPM2 (M2), MPM3 (M3) of *P. arizonense* genome by conventional PCR analysis. M is 100

molecular weight ladder strand. The samples were loaded on two separated gels, and the edges were processed in parallel.

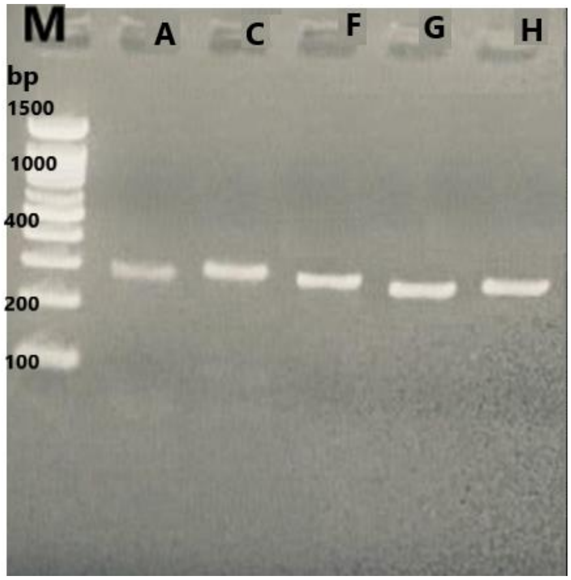

Fig. S. (3): Gel electrophoresis of RT-PCR products of MPA gen cluster, *mpaA* (lane A*)*, *mpaC* (lane C), *mpaF* (lane F), *mpaG* (lane G), *mpaH* (lane H), extracted from MPA overproducer mutant strains *P*. *arezonense* HE-MPM2.

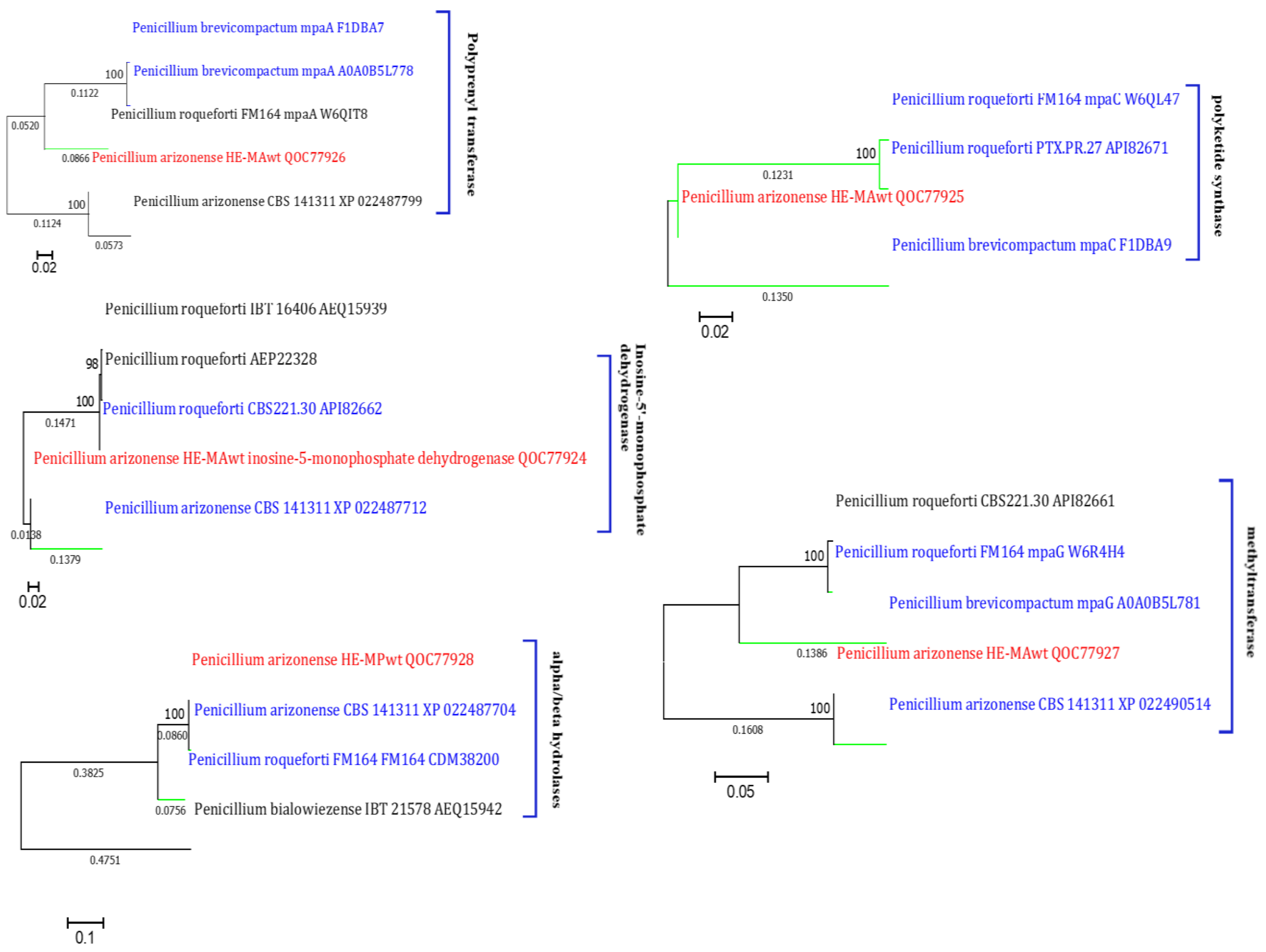

Fig. S. (4) Phylogenetic analysis of putative protein in *P. arizonense* HE-MPwt responsible for MPA biosynthesis gene clusters with closely related protein of Penicillium strains retrieved from NCBI GenBank. The analysis was conducted by constructing a rooted tree using the UPGMA method in the MEGA7 program. The percentage of replicate trees in which the associated taxa clustered together in the bootstrap test (1000 replicates) is shown next to each branch.

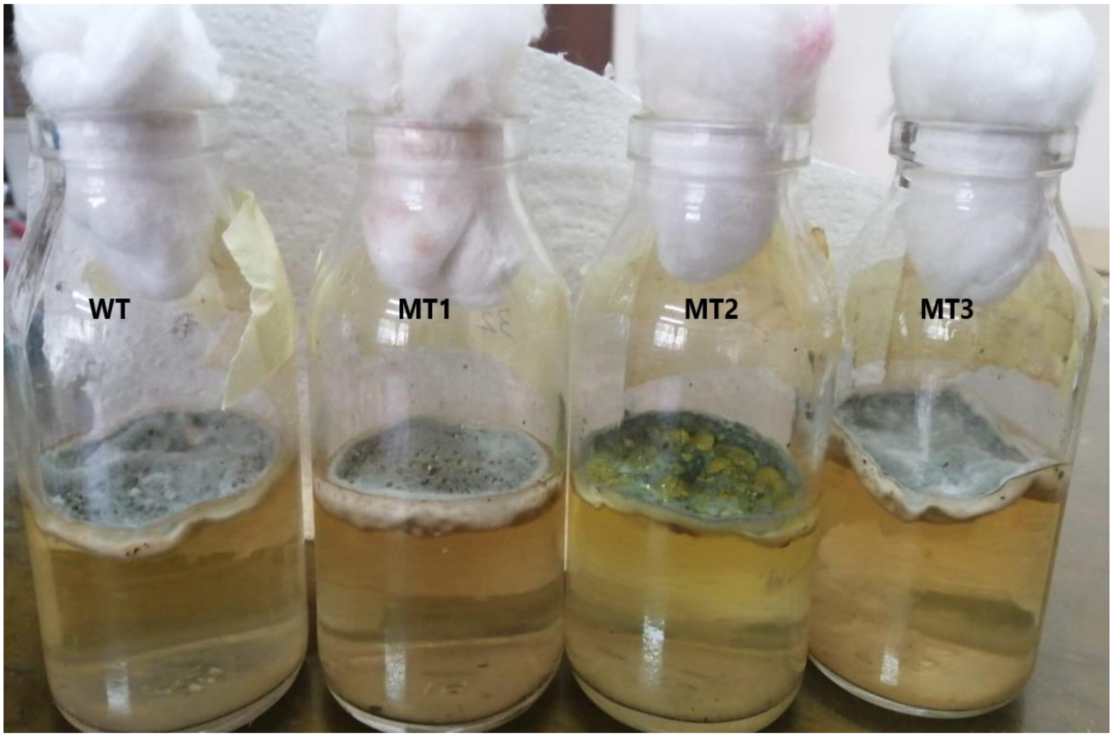

Fig. S. (5) Morphological behaviors of wild type (WT), and mutant strains (MT1, MT2, and MT3) of *P. arizonense*-HE after 7 d of growth on PD broth culture medium and incubated at 25 ^{0}C.

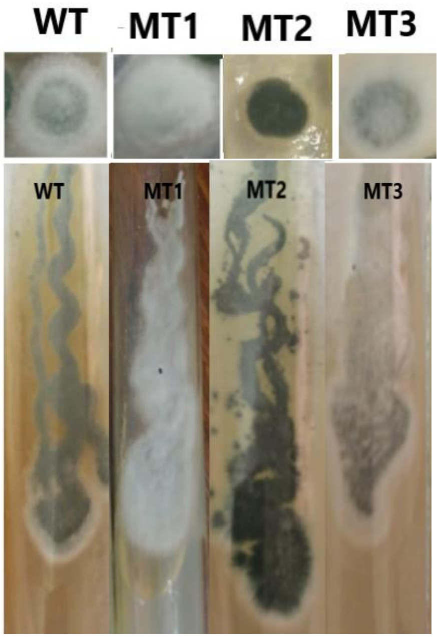

Fig. S. (6) Morphological behaviors of wild type (WT), and mutant strains (MT1, MT2, and MT3) of *P. arizonense*-HE grew on PD plate and slants culture medium after 5 d of incubation at 25 ^{o}C.
